# Suspected acquired factor XIII deficiency in a man living with HIV: diagnostic and therapeutic challenges in recurrent spontaneous hemorrhage: a case report

**DOI:** 10.3389/fmed.2026.1768171

**Published:** 2026-05-22

**Authors:** Wei He, Juecai Chen, Hongwei Wu

**Affiliations:** Department of Hematology, The First Affiliated Hospital of Chengdu Medical College, Chengdu, China

**Keywords:** acquired factor XIII deficiency, HIV, immunosuppressive therapy, infection, replacement therapy, spontaneous hemorrhage

## Abstract

Factor XIII (FXIII) deficiency is a rare yet potentially life-threatening bleeding disorder, which typically manifests with normal results on routine coagulation assays: a key factor contributing to its frequent underdiagnosis. People living with HIV (PLWH) are predisposed to hemostatic abnormalities; however, the coexistence of HIV infection and a possible acquired FXIII deficiency has been rarely documented in the literature. Herein, we describe a rare case of suspected acquired FXIII deficiency in an HIV-positive man with a well-controlled infection on long-term antiretroviral therapy (ART), who presented with recurrent spontaneous bleeding that led to spontaneous splenic rupture and delayed postoperative hemorrhage. A clot solubility test (CST) demonstrated complete dissolution of the fibrin clot within 24 h, indicating the possibility of FXIII deficiency. Based on clinical manifestations and limited laboratory data, a diagnosis of suspected acquired FXIII deficiency was formulated. In resource-limited settings, a pragmatic management strategy encompasses on-demand cryoprecipitate replacement, and meticulous perioperative planning. Immunosuppressive therapy was administered in consideration of a potential immune-mediated etiology, yet this intervention failed to resolve the patient’s recurrent spontaneous bleeding.

## Introduction

Factor XIII (FXIII) mediates fibrin cross-linkage at the final step of the coagulation cascade, thereby stabilizing hemostatic clots and preventing premature lysis (1). Acquired FXIII deficiency is a rare bleeding disorder characterized by decreased catalytic activity. Its etiologies are generally classified into non-immune causes and immune-mediated forms, the latter being driven by specific autoantibodies that neutralize FXIII function or accelerate its clearance (2–4). Affected patients typically present with severe, delayed, and potentially life-threatening bleeding, which represents a substantial clinical challenge.

Diagnosis of this disorder is challenging, as routine coagulation screening tests, including prothrombin time (PT) and activated partial thromboplastin time (APTT), typically yield normal results. Therefore, it is critical for clinicians to maintain a high index of suspicion based on characteristic bleeding manifestations. Definitive diagnosis requires specific FXIII activity assays. The clot solubility test (CST) is merely a screening indicator for compromised clot stability, lacking specificity for FXIII deficiency; an abnormal CST may also result from hyperfibrinolysis and other hemostatic disorders.

Human immunodeficiency virus (HIV) infection is frequently associated with complex hemostatic abnormalities, and most existing studies have focused on thrombotic complications in this population. Nevertheless, HIV-associated hemostatic dysfunction can present with divergent phenotypes, and bleeding complications have long been overlooked compared with thrombosis. In contrast, reports of rare bleeding disorders, especially acquired FXIII deficiency complicated with HIV infection, remain extremely rare, with limited data available regarding their clinical characteristics, diagnostic dilemmas, and management strategies. Chronic immune activation and immune dysregulation related to HIV infection may contribute to the production of FXIII autoantibodies, although the exact pathophysiological link between them remains unclear. To the best of our knowledge, this is the first detailed case report of suspected acquired FXIII deficiency in an HIV-infected patient with long-term antiretroviral therapy (ART) and virologic suppression. This case systematically illustrates the diagnostic and therapeutic procedures in resource-limited settings, aiming to raise clinicians’ awareness of rare bleeding disorders in HIV-infected patients and promote further research into their underlying pathophysiological connections.

## Case description

A 69-year-old man with a 10-year history of HIV infection was maintained on long-term suppressive ART with efavirenz, lamivudine, and tenofovir disoproxil fumarate. His HIV RNA levels remained undetectable, and his CD4^+^ T-lymphocyte count was consistently above 500 cells/μL (most recent value: 512 cells/μL) throughout the premorbid period. He had no personal or family history of spontaneous bleeding, coagulation disorders, liver disease, or autoimmune disease.

In September 2019, he was admitted to our hospital for a painful swelling in the right lower extremity that developed one week after a minor fall. On physical examination, vital signs were stable. No jaundice was evident in the skin, mucous membranes, and sclerae. No superficial lymphadenopathy was palpable. A tender, ecchymotic mass measuring approximately 6 × 8 cm was noted over the right thigh, with multiple ecchymoses scattered across the extremities and abdominal wall. Despite obvious bleeding manifestations, routine coagulation parameters were within normal limits: PT 12.3 s (10.0–14.0 s), APTT 34.6 s (25.0–38.0 s), thrombin time (TT) 15.6 s (10.3–16.6 s), fibrinogen 3.2 g/L (2.0–4.0 g/L), D-dimer 229 ng/mL (0–243 ng/mL), fibrin-fibrinogen degradation products (FDP) 3.1 μg/mL (0–5.0 μg/mL), and platelet count 216 × 10^9^/L (100–300 × 10^9^/L). The hematoma resolved completely after conservative management, and the patient was discharged.

Over the following years, he experienced recurrent, multifocal spontaneous bleeding episodes. The timeline, bleeding sites and relevant treatments are summarized in Table 1. On each presentation, he had prominent hemorrhagic manifestations, whereas serial routine coagulation tests (including PT, APTT, TT, fibrinogen, D-dimer, FDP, and platelet count) remained consistently within the normal range. This marked discrepancy between the clinical bleeding phenotype and standard coagulation results prompted further investigation. After reviewing the literature, FXIII deficiency was clinically suspected. CST adopted as a screening tool for clot instability, revealed complete fibrin clot lysis within 24 h. The abnormal CST result thus strongly raised suspicion of potential FXIII deficiency.

**Table 1:** Chronological summary of clinical bleeding episodes and interventions.

Date	Key clinical events	Additional examinations	Key interventions
Sep 2019	Post-traumatic hematoma (right thigh)	Ultrasound	FFP
Mar 2020	Hematoma (left lower extremity)	MRI	FFP
Apr 2020	Multifocal hematomas (shoulder, chest wall, abdomen)	CT	FFP
Aug 2020	Hematoma (right upper arm)	Ultrasound	FFP (allergic reaction)^†^; Methylprednisolone; Cyclophosphamide
Nov 2020	Hematomas (left upper arm and left gluteal region)	CT and MRI	Cryoprecipitate
Jan 2021	Hematoma (left gluteal region)	CT	Cryoprecipitate
Apr 2021	Massive hematoma (lumbar/abdominal region)	CT	Cryoprecipitate
Jan 2022	Hematomas (right neck and right upper arm)	CT and Ultrasound	Cryoprecipitate
May 2022	Hematoma (chest wall)	CT	Cryoprecipitate
Sep 2022	Hematoma (left neck/shoulder region)	CT	Cryoprecipitate
Mar 2023	Hematoma (right axilla)	Ultrasound	Cryoprecipitate
May 2023	Hematoma (left forearm)	Ultrasound	Cryoprecipitate
Jun 2023	Splenic rupture	CT	Cryoprecipitate
Mar 2025	Hematoma (left forearm) and Hemorrhage(urinary tract)	Ultrasound and Urinalysis	Cryoprecipitate

FFP, fresh frozen plasma; CT, computed tomography; MRI, magnetic resonance imaging.

^†^The patient developed a severe allergic reaction (dyspnea and generalized pruritus) during FFP infusion, leading to its discontinuation and a permanent switch to cryoprecipitate for all subsequent transfusions.

To exclude other common causes of bleeding, further investigations were performed. Liver function tests, including albumin, transaminases, and bilirubin, were normal. Serologic screening for autoimmune diseases, including antinuclear antibodies and rheumatoid factor, was negative. Apart from his ART, the patient was not taking any other medications known to impair coagulation. An extensive workup for malignancy, including computed tomography scans of the chest and abdomen as well as serum tumor marker testing, was also performed and revealed no evidence of neoplastic disease. Combined with the abnormal CST findings indicating FXIII dysfunction and the exclusion of alternative bleeding causes, acquired FXIII deficiency was clinically suspected. Definitive laboratory confirmation could not be achieved, as FXIII activity, antigen, and inhibitor assays were unavailable in our hospital.

Initially, the patient received fresh frozen plasma (FFP) as replacement therapy during bleeding episodes, with prompt clinical improvement. However, FFP failed to prevent recurrent spontaneous hemorrhages, and a severe allergic reaction occurred during one infusion. Replacement therapy was subsequently switched to cryoprecipitate. With cryoprecipitate, acute bleeding symptoms could be controlled, but recurrent bleeding persisted. Given the underlying HIV infection and the suboptimal response to replacement therapy, immune-mediated FXIII deficiency was suspected.

To suppress the presumed production of FXIII inhibitors, immunosuppressive therapy was initiated: methylprednisolone at a dose of 40 mg/day for 1 week, followed by a gradual taper to 10 mg/day over 4 weeks, in combination with cyclophosphamide at 50 mg/day for 8 weeks. Despite this therapeutic regimen, there was no significant reduction in the frequency of bleeding episodes. In light of the limited efficacy and concerns regarding an elevated risk of opportunistic infections in the setting of HIV infection, immunosuppressive therapy was discontinued after 3 months.

**Figure 1:**
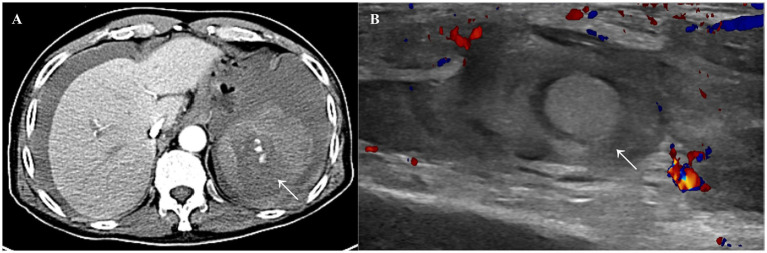
**(A)** Contrast-enhanced abdominal CT revealed splenic rupture complicated by hemoperitoneum. **(B)** Ultrasound examination suggested a hypoechoic mass in the left forearm, with a size of approximately 2.6 × 2.2 × 4.6 cm, which was considered to be a hematoma.

In June 2023, the patient presented with acute abdominal pain. Contrast-enhanced computed tomography revealed splenic rupture (Figure 1A), which was considered spontaneous in the absence of trauma. He underwent emergency splenectomy. On postoperative day 3, he required reoperation for persistent wound oozing, reflecting the characteristic delayed postoperative hemorrhage seen in suspected FXIII deficiency. Bleeding was controlled with perioperative cryoprecipitate transfusion.

In March 2025, he was readmitted with swelling and pain in the upper extremity (Figure 1B), accompanied by gross hematuria. His symptoms improved following cryoprecipitate replacement therapy, and he was discharged in stable condition. Since that admission, he has not required further hospitalization for spontaneous bleeding, under a strategy of on-demand cryoprecipitate transfusion and avoidance of high-risk interventions.

## Discussion

This case is notable for the age of onset (>60 years), the severe and multifocal bleeding phenotype, the persistent discordance between clinical manifestations and routine coagulation tests, and the diagnostic and therapeutic constraints in a resource-limited setting. To our knowledge, the coexistence of HIV infection and acquired FXIII deficiency has not been previously reported, particularly in a virologically suppressed patient with relatively preserved CD4^+^ T-cell counts.

FXIII deficiency is a rare disorder that predominantly affects older adults and is characterized by potentially life-threatening spontaneous bleeding, including intracranial hemorrhage, visceral bleeding, and extensive soft tissue hematomas (5). These features are fully reflected in our patient, who presented with recurrent spontaneous hematomas over several years and ultimately developed spontaneous splenic rupture, followed by delayed wound oozing after splenectomy. The latter event is highly characteristic of suspected FXIII deficiency, in which initial hemostasis may appear adequate, but fibrin clots are structurally unstable and susceptible to premature lysis, leading to delayed postoperative or post-traumatic bleeding.

In clinical practice, FXIII deficiency is easily misdiagnosed or overlooked. Routine hemostatic parameters, such as PT, APTT, TT, D-dimer, fibrinogen, FDP, and platelet count, are typically normal because FXIII acts at the final stage of the coagulation cascade. As a result, a high index of clinical suspicion is a prerequisite for diagnosis, particularly in patients with recurrent spontaneous bleeding or delayed hemorrhage after surgery in the setting of normal screening tests. Specialized laboratory investigations are required for definitive confirmation. The CST is a simple and inexpensive qualitative assay available in many laboratories, and it raised high clinical suspicion in our patient. However, it is important to recognize that the CST is a screening test for impaired clot stability rather than a specific diagnostic test for FXIII deficiency, as abnormal results may also occur in hyperfibrinolytic disorders, including alpha-2-antiplasmin deficiency and plasminogen activator inhibitor-1 (PAI-1) deficiency (6). Moreover, its sensitivity is low and it detects only severe FXIII deficiency (activity <0.02 U/mL) (7, 8). Quantitative assays for FXIII activity (FXIII: Act) and antigen (FXIII: Ag) are more informative for diagnosing and monitoring both severe and milder forms of FXIII deficiency (4). In addition, inhibitor (autoantibody) testing can help differentiate congenital deficiency from autoantibody-mediated acquired FXIII deficiency (9).

Given the patient’s typical bleeding manifestations, we conducted stepwise clinical reasoning. First, routine coagulation tests were unremarkable and failed to explain his distinctive bleeding phenotype. Second, a normal D-dimer level ruled out severe systemic hyperfibrinolysis. Third, we excluded common secondary bleeding triggers, including hepatic dysfunction, autoimmune diseases, drug-induced hemostatic impairment and malignancy. Finally, an abnormal CST indicated impaired fibrin clot stability. However, several critical laboratory tests could not be performed due to limited local laboratory resources. The euglobulin clot lysis time (ECLT) assay was unavailable to exclude mild or localized hyperfibrinolysis. Similarly, quantitative FXIII measurement and inhibitor testing were not conducted to confirm the diagnosis or distinguish between immune and non-immune pathogenic mechanisms. Integrating all clinical manifestations and laboratory findings, we tentatively diagnosed suspected FXIII deficiency.

Because acquired FXIII deficiency is extremely rare, the optimal treatment regimen remains poorly defined (10), and no unified treatment guidelines have been established nationwide or worldwide (11). Replacement therapy is the cornerstone of the management of acute bleeding. Available options include FFP, cryoprecipitate, plasma-derived FXIII concentrates (such as Corifact/Fibrogammin P), and recombinant FXIII-A₂ concentrate (Tretten) (12). Cryoprecipitate is frequently used in routine practice because it contains higher FXIII levels (approximately 3 IU/mL) than FFP (approximately 1 IU/mL) (13). Plasma-derived and recombinant FXIII concentrates offer advantages of higher purity, standardized dosing, and a lower risk of transfusion-transmitted infection, yet such agents remain unavailable in many low- and middle-income countries.

Our patient initially received FFP with a good hemostatic response, but developed a severe allergic reaction, necessitating a switch to cryoprecipitate. Thereafter, bleeding episodes, including emergent surgery for splenic rupture, were successfully managed with on-demand cryoprecipitate replacement, although recurrent bleeding persisted. This experience underscores both the feasibility and limitations of cryoprecipitate-based transfusion in resource-constrained settings: acute bleeding can be controlled, but repeated transfusions are required, precise FXIII dosing is difficult, and long-term prophylaxis is usually impractical.

Only a suspected acquired FXIII deficiency was diagnosed in this case, with no clear distinction between immune-mediated and non-immune-mediated pathogenesis. In consideration of the patient’s underlying HIV infection, we presume that the occurrence of FXIII deficiency is potentially linked to HIV infection. In fact, HIV infection exerts extensive impacts on the coagulation system, and its dysregulated hemostatic balance has been well addressed in previous studies. Such hemostatic dysregulation is collectively mediated by chronic immune activation, persistent systemic inflammation and progressive endothelial dysfunction (14), which substantially elevates the risks of venous thromboembolism and arterial cardiovascular events (15, 16). The mechanisms underlying HIV-associated coagulation abnormalities are not fully elucidated and may involve host-related factors, antiretroviral medications, viral effects, and other confounders (17). In contrast to the well-documented hypercoagulable diathesis in PLWH, the occurrence of HIV infection and acquired FXIII deficiency has not been previously reported, highlighting the novelty of the present case.

The potential pathophysiological mechanisms linking HIV infection to acquired FXIII deficiency are mainly speculative but deserve focused discussion. Chronic immune activation and persistent immune dysregulation, which are characteristic of HIV infection even during effective ART, may disrupt immune tolerance and trigger the production of autoantibodies against FXIII, which may partly explain the declined FXIII activity in this patient. Previous studies have reported that autoantibodies against FXIII could affect its bioactivity: neutralizing antibodies impair FXIII function by inhibiting the clearance of activation peptides and interfering with FXIII cross-linking to fibrin, whereas non-neutralizing antibodies act by accelerating FXIII clearance (18, 19). Impaired hepatic synthetic function represents another plausible pathway. Direct effects of HIV on hepatocytes, or mild hepatotoxicity associated with certain antiretroviral agents, could theoretically suppress coagulation factor synthesis. While direct evidence is limited, studies have shown that compromised hepatocellular function, such as under hypoxic conditions, can impair translation of the FXIII-B subunit, potentially leading to reduced circulating FXIII levels (20). Furthermore, accelerated consumption secondary to chronic inflammation may serve as an additional contributing factor. The sustained activation of the coagulation cascade in the setting of chronic HIV-related inflammation may result in accelerated consumption of coagulation factors including FXIII. This phenomenon mirrors acquired, consumption-related FXIII deficiency observed in patients with severe trauma, major surgery, inflammatory bowel disease, and sepsis (21). Notably, the patient achieved complete virological suppression with preserved CD4^+^ T-cell counts, indicating that advanced immunodeficiency is not a prerequisite for this complication. Even clinically stable HIV-infected individuals may develop rare bleeding disorders with reduced FXIII activity due to chronic immune activation and sustained inflammation.

For immune-mediated acquired FXIII deficiency, immunosuppressive therapy is recommended to eliminate autoantibodies. First-line regimens typically consist of corticosteroids combined with cyclophosphamide or rituximab, while second-line options include mycophenolate mofetil or cyclosporine A (22). Based on our center’s experience with this regimen, its cost-effectiveness, and its favorable local accessibility compared with other agents such as rituximab, a combination of corticosteroids and cyclophosphamide was ultimately selected for this patient. In addition, therapeutic approaches such as immunoadsorption, plasma exchange, and immunoglobulin infusion can also be employed to reduce inhibitor levels, improve FXIII activity, and control bleeding symptoms in patients (22). Currently, there is no evidence to support the prophylactic infusion of FXIII-containing blood products or formulations for immune-mediated acquired FXIII deficiency. However, relevant literature has indicated that the dosage of FXIII prophylactic therapy for such patients is typically higher and administered more frequently than that for patients with hereditary FXIII deficiency (23). Reported response rates to these regimens are highly variable and likely reflect the heterogeneity of autoantibodies and underlying immune mechanisms (24–26). In the present case, a combination of methylprednisolone and low-dose cyclophosphamide failed to significantly reduce bleeding frequency. Given the lack of clear benefit and the elevated risk of opportunistic infections, immunosuppressive therapy was discontinued after 3 months. This highlights the need for individualized treatment decisions based on bleeding severity, suspected pathophysiology, comorbidities and local medical resources. For PLWH in particular, clinicians must carefully balance the benefits of immunosuppressive therapy against relevant risks. This clinical consideration applies not only to conventional immunosuppressants but also to other immunomodulatory approaches. Accumulating evidence shows that immune checkpoint inhibitors (ICIs) for malignancies in PLWH also require strict safety assessment. Fortunately, these agents are generally well tolerated in patients with sustained viral suppression and intact immune function (27, 28). We are also considering switching to rituximab or other alternative immunosuppressive strategies for subsequent treatment.

This report has several limitations. First, specific assays for hyperfibrinolysis, such as the ECLT, were unavailable at our institution. Additionally, quantitative FXIII activity and antigen testing, as well as FXIII inhibitor assays, were not performed. For these reasons, our diagnosis of suspected acquired FXIII deficiency remains provisional, established on the basis of clinical bleeding manifestations, a positive CST result, and exclusion of common differentials, rather than definitive laboratory confirmation. The late onset of bleeding, negative family history, and absence of lifelong bleeding symptoms make severe congenital FXIII deficiency highly unlikely, although confirmatory genetic testing was not conducted due to financial constraints. Finally, as a single case report, our observations cannot establish a causal relationship between HIV infection and suspected FXIII deficiency, nor can they define optimal therapy. In summary, this case represents a preliminary presumptive diagnosis of suspected acquired FXIII deficiency formulated within a resource-limited setting, and our conclusions are therefore expressed with appropriate caution.

Despite these limitations, this case adds to the limited body of evidence regarding suspected acquired FXIII deficiency and, to the best of our knowledge, represents the first report of its occurrence in a patient with well-controlled HIV infection. It provides several practical messages for clinicians. First, FXIII deficiency should be considered in patients presenting with recurrent spontaneous bleeding or delayed postoperative hemorrhage, particularly when PT, APTT, fibrinogen, and platelet counts are all within normal limits. Second, in PLWH, bleeding manifestations should not be attributed solely to thrombocytopenia, liver disease, or drug effects; rare coagulation factor disorders must also be kept in mind. Third, even in resource-limited settings without access to FXIII concentrates, quantitative FXIII assays, or inhibitor testing, the combination of CST, meticulous clinical assessment, and on-demand cryoprecipitate replacement may provide a practical, albeit imperfect, clinical management approach.

## Conclusion

This case report describes the diagnostic and therapeutic management of an HIV-positive patient who presented with recurrent spontaneous bleeding secondary to provisionally suspected acquired FXIII deficiency. The findings presented here chiefly highlight the unique diagnostic dilemmas of rare bleeding disorders in resource-limited clinical settings, with restricted access to specialized coagulation assays. No definitive causal relationship between HIV infection and acquired FXIII deficiency can be established in this case, as critical confirmatory laboratory tests are lacking. The previously mentioned pathogenic mechanisms associated with HIV remain merely speculative and unproven, and should not be overemphasized. Further clinical research is urgently required to optimize diagnostic confirmation methods, standardize screening procedures, and refine individualized management strategies for patients with suspected FXIII deficiency, so as to address the common diagnostic bottlenecks in under-resourced medical centers.

## Data Availability

The raw data supporting the conclusions of this article will be made available by the authors, without undue reservation.

## References

[1] Muszbek L Bereczky Z Bagoly Z Komáromi I Katona É. Factor XIII: a coagulation factor with multiple plasmatic and cellular functions. Physiol Rev. (2011) 91:931–72. doi: 10.1152/physrev.00016.2010, 21742792

[2] Ichinose A. Autoimmune acquired factor XIII deficiency due to anti-factor XIII/13 antibodies: a summary of 93 patients. Blood Rev. (2017) 31:37–45. doi: 10.1016/j.blre.2016.08.002, 27542511

[3] Biswas A Ivaskevicius V Thomas A Oldenburg J. Coagulation factor XIII deficiency. Diagnosis, prevalence and management of inherited and acquired forms. Hamostaseologie. (2014) 34:160–6. doi: 10.5482/HAMO-13-08-0046, 24503678

[4] Kohler HP Ichinose A Seitz R Ariens RAS Muszbek L. Diagnosis and classification of factor XIII deficiencies. J Thromb Haemost. (2011) 9:1404–6. doi: 10.1111/j.1538-7836.2011.04315.x, 22946956

[5] Kessel R Hu C Shore-Lesserson L Rand J Manwani D. A child with acquired factor XIII deficiency: case report and literature review. Haemophilia. (2013) 19:814–26. doi: 10.1111/hae.12145, 23607876

[6] Cheves TA DeMarinis S Sweeney JD. Laboratory methods in the assessment of hereditary hemostatic disorders. Hematol Oncol Clin North Am. (2021) 35:1051–68. doi: 10.1016/j.hoc.2021.07.002 34391602

[7] Jennings I Kitchen S Menegatti M Palla R Walker I Makris M . Detection of factor XIII deficiency: data from multicentre exercises amongst UK NEQAS and PRO-RBDD project laboratories. Int J Lab Hematol. (2017) 39:350–8. doi: 10.1111/ijlh.12633, 28406553

[8] Hsu P Zantek ND Meijer P Hayward CP Brody J Zhang X . Factor XIII assays and associated problems for laboratory diagnosis of factor XIII deficiency: an analysis of international proficiency testing results. Semin Thromb Hemost. (2014) 40:232–8. doi: 10.1055/s-0034-1365841, 24497117

[9] Karimi M Peyvandi F Naderi M Shapiro A. Factor XIII deficiency diagnosis: challenges and tools. Int J Lab Hematol. (2018) 40:3–11. doi: 10.1111/ijlh.12756, 29027765

[10] Boehlen F Casini A Chizzolini C Mansouri B Kohler HP Schroeder V . Acquired factor XIII deficiency: a therapeutic challenge. Thromb Haemost. (2013) 109:479–87. doi: 10.1160/TH12-08-0604, 23306660

[11] Muszbek L Katona E. Diagnosis and management of congenital and acquired FXIII deficiencies. Semin Thromb Hemost. (2016) 42:429–39. doi: 10.1055/s-0036-1572326, 27071048

[12] Inbal A Oldenburg J Carcao M Rosholm A Tehranchi R Nugent D. Recombinant factor XIII: a safe and novel treatment for congenital factor XIII deficiency. Blood. (2012) 119:5111–7. doi: 10.1182/blood-2011-10-386045, 22451421

[13] Wakabayashi N Nishioka H Yuzuriha S. Recurrent bleeding after head trauma caused by acquired factor XIII deficiency. Plast Reconstr Surg Glob Open. (2022) 10:e4109. doi: 10.1097/GOX.0000000000004109, 35186643 PMC8846273

[14] Hetta HF Alanazi FE Alshareef H Alqifari SF Bukhari SQ Albalwi MA . Antiviral drugs in HIV and cardiovascular disease: mechanistic insights and clinical implications. Pharmaceuticals. (2026) 19:205. doi: 10.3390/ph19020205, 41754747 PMC12943153

[15] Shen YM Frenkel EP. Thrombosis and a hypercoagulable state in HIV infected patients. Clin Appl Thromb Hemost. (2004) 10:277–80. doi: 10.1177/107602960401000311, 15247986

[16] Subramanya V McKay HS Brusca RM Palella FJ Kingsley LA Witt MD . Inflammatory biomarkers and subclinical carotid atherosclerosis in HIV-infected and HIV-uninfected men in the Multicenter AIDS cohort study. PLoS One. (2019) 14:e0214735. doi: 10.1371/journal.pone.0214735, 30946765 PMC6448851

[17] Snopkova S Matyskova M Havlickova K Jarkovsky J Svoboda M Zavrelova J . Increasing procoagulant activity of circulating microparticles in patients living with HIV. Med Mal Infect. (2020) 50:555–61. doi: 10.1016/j.medmal.2019.09.013 31611134

[18] Souri M Osaki T Ichinose A. Anti-factor XIII a subunit (FXIII-A) autoantibodies block FXIII-A2 B2 assembly and steal FXIII-A from native FXIII-A2B2. J Thromb Haemost. (2015) 13:802–14. doi: 10.1111/jth.12877, 25703841

[19] Souri M Ozawa T Osaki T Koyama T Muraguchi A Ichinose A. Cloning of human antifactor XIII monoclonal antibody dissects mechanisms of polyclonal antibodies in a single patient. J Thromb Haemost. (2023) 21:255–68. doi: 10.1016/j.jtha.2022.11.019, 36700504

[20] Hettiarachchi GK Katneni UK Hunt RC Kames JM Athey JC Bar H . Translational and transcriptional responses in human primary hepatocytes under hypoxia. Am J Physiol Gastrointest Liver Physiol. (2019) 316:G720–34. doi: 10.1152/ajpgi.00331.2018, 30920299 PMC6620582

[21] Yan MTS Rydz N Goodyear D Sholzberg M. Acquired factor XIII deficiency: a review. Transfus Apher Sci. (2018) 57:724–30. doi: 10.1016/j.transci.2018.10.013, 30446212

[22] Smith J Bodine JS Cunningham MT Gooley K Plapp FV Dasgupta A . Perioperative therapeutic plasma exchange in a patient with rare factor XIII inhibitor. Transfus Apher Sci. (2023) 62:103654. doi: 10.1016/j.transci.2023.103654, 36775674

[23] Ogawa Y. Acquired autoimmune coagulation factor XIII/13 deficiency. Rinsho Ketsueki. (2020) 61:799–808. doi: 10.11406/rinketsu.61.799, 32759568

[24] Schött U Astermark J Zdanowski A Strandberg K. Factor XIII deficiency-not only a congenital bleeding disorder. Lakartidningen. (2023) 26:23018. 37099358

[25] Tone KJ James TE Fergusson DA Tinmouth A Tay J Avey MT . Acquired factor XIII inhibitor in hospitalized and perioperative patients: a systematic review of case reports and case series. Transfus Med Rev. (2016) 30:123–31. doi: 10.1016/j.tmrv.2016.04.001, 27167905

[26] Muszbek L Pénzes K Katona É. Auto-and alloantibod-ies against factor XIII: laboratory diagnosis and clinical consequences. J Thromb Haemost. (2018) 16:822–32. doi: 10.1111/jth.13982.26 29460500

[27] Hetta HF Alatawi Y Alanazi FE Alattar A Alshaman R Alshareef H . Evaluating the safety and efffcacy of PD-1 inhibitors in HIV patients diagnosed with lung cancer: a systematic review. Pharmaceuticals. (2025) 18:1654. doi: 10.3390/ph18111654 41304899 PMC12655231

[28] Alatawi AD Alaqy AB Alalawi RJ Alqarni RS Sufyani RA Alqarni GS . Safety and efffcacy of immune checkpoint inhibitors in human immunodeffciency virus-associated cancer: a systematic scoping review. Diseases. (2025) 13:230. doi: 10.3390/diseases13080230.28 40863204 PMC12385830

